# Delayed isolation of smear-positive pulmonary tuberculosis patients in a Japanese acute care hospital

**DOI:** 10.1186/s12890-018-0653-1

**Published:** 2018-05-31

**Authors:** Sho Nishiguchi, Shusaku Tomiyama, Izumi Kitagawa, Yasuharu Tokuda

**Affiliations:** 1 Department of General Internal Medicine, Shonan Kamakura General Hospital, 1370-1 Okamoto, Kamakura, Kanagawa Japan; 2 Department of General Medicine, Iizuka Hospital, Fukuoka, Japan; 3 Muribushi Okinawa Project for Teaching Hospitals, Okinawa, Okinawa Prefecture Japan; 4 Department of Internal Medicine, Hayama Heart Center, Hayama, Kanagawa Japan; 5 Unit of Public Health and Preventive Medicine, School of Medicine, Yokohama City University, Yokohama, Kanagawa Japan

**Keywords:** Pulmonary tuberculosis, Delay, Isolation, Diagnosis, Smear-positive

## Abstract

**Background:**

Active pulmonary tuberculosis (TB) is associated with intra-hospital spread of the disease. Expeditious diagnosis and isolation are critical for infection control. However, factors that lead to delayed isolation of smear-positive pulmonary TB patients, especially among the elderly, have not been reported. The purpose of this study is to investigate factors associated with delay in the isolation of smear-positive TB patients.

**Methods:**

All patients with smear-positive pulmonary TB admitted between January 2008 and December 2016 were included. The setting was a Japanese acute care teaching hospital. Following univariate analysis, significant factors in the model were analyzed using the multivariate Cox proportional hazard model.

**Results:**

Sixty-nine patients with mean age of 81 years were included. The median day to the isolation of pulmonary TB was 1 day with interquartile range, 1–4 days. On univariate analysis, the time to isolation was significantly delayed in male patients (*p* = 0.009), in patient who had prior treatment with newer quinolone antibiotics (*p* = 0.027), in patients who did not have chronic cough (*p* = 0.023), in patients who did not have appetite loss (*p* = 0.037), and in patients with non-cavitary lesion (*p* = 0.005), lesion located other than in the upper zone (*p* = 0.015), and non-disseminated lesion on the chest radiograph (*p* = 0.028). On multivariate analysis, the time to isolation was significantly delayed in male patients (hazard ratio [HR], 0.47; 95% confidence interval [CI], 0.25 to 0.89; *P* = 0.02), in patients who did not have chronic chough (HR, 0.52; 95% CI, 0.28 to 0.95; *P* = 0.033), and in patients with non-cavitary lesion on the chest radiograph (HR, 0.46; 95% CI, 0.23 to 0.92; *P* = 0.028).

**Conclusions:**

In acute care hospitals of an aging society, prompt diagnosis and isolation of TB patients are important for the protection of other patients and healthcare providers. Delay in isolation is associated with male gender, absence of chronic cough, and presence of non-cavitary lesions on the chest radiograph.

## Background

Tuberculosis (TB) control is of worldwide interest, especially among patients with active pulmonary TB who are smear-positive for acid-fast bacilli [1]. TB control requires early diagnosis and immediate initiation of treatment [2]. Delay in diagnosis adversely affects clinical outcome; besides, it increases transmission within the community and leads to higher transmission rates in an epidemic [3, 4].

Although the prevalence of TB had reduced to 16.7 per 100,000 among Japanese population in 2012, this figure remains 3–4 times higher than in Europe and North America [5, 6]. One reason for the relatively high prevalence of TB in Japan is an aging population with previous TB infection [7]. In the World Health Organization 2017 global report, Japanese patients with smear-positive pulmonary TB were older and had a higher mortality than in other countries [8, 9]. Given the high rates of hospital admission among elderly patients in developed countries, any delay in diagnosis and isolation of pulmonary TB can cause spread of infection [10]. Therefore, in aging societies including Japan, it is important to seek reasons for delay in the isolation of pulmonary TB patients.

In previous studies, factors leading to delayed diagnosis of pulmonary TB have been well reported worldwide [2, 11–14]. These reports focused on ‘patient delay’ and ‘health care system delay’ [14–16]. Delayed diagnosis has been associated with human immunodeficiency virus infection, chronic cough, and presence of incidental lung disease [2]. Geographical and socio-psychological barriers include rural residence and poor access to healthcare [2, 11, 12]. Other factors that may delay diagnosis include old age, female gender, alcoholism and substance abuse, initial visitation at a low-level government healthcare facility, private practitioner, or traditional healer [2, 11, 12, 14]. History of immigration, poor education status, low awareness of TB, irrational beliefs, self-treatment, the associated stigma, consultation at a public hospital, and prior treatment with fluoroquinolones may also delay the diagnosis of active pulmonary TB [2, 11–14].

To the best of our knowledge, factors associated with delay in isolation of pulmonary TB patients have not been reported. In the clinical setting, any delay in isolation is critical to control of infection within hospitals. Therefore, we conducted this study to clarify factors associated with delayed isolation of active pulmonary TB patients.

## Methods

### Patients and setting

We conducted a retrospective study on patients who were diagnosed with smear-positive pulmonary TB in an urban teaching hospital between January 2008 and December 2016. The hospital provides primary through tertiary care in Kanagawa Prefecture, Japan, with a population of about nine million people. The hospital has no specific wards for patients with treatment of active pulmonary TB. Those who are suspected to have active pulmonary TB disease are immediately isolated in single rooms with negative air pressure for definite sputum smear diagnosis, particularly with three consecutive sputum or gastric aspiration analysis. After the diagnosis of smear-positive pulmonary TB is established, the patients are then transferred to another hospital that has special wards for the treatment of smear-positive pulmonary TB. Therefore, a delay in isolation on admission may spread the TB infection in-hospital. We studied 69 consecutive patients who were diagnosed with smear-positive pulmonary TB by examination of the sputum or gastric specimen.

### Data collection

Data collection for each patient, collectively referred to as “patient-related factors”: age, gender, history of smoking or alcoholism, welfare recipient or not, activities of daily living, past history of TB, contact with TB patients, diabetes mellitus, other lung disease, surgical history of gastric resection, human immunodeficiency virus infection, preceding treatment with newer quinolone antibiotics, steroid use, chronic cough, loss of appetite, body mass index, body temperature on arrival, systolic blood pressure, oxygen requirement, hemoglobin level, serum albumin level, C-reactive protein level, presence of a cavitary, upper zone or disseminated lesion on the chest radiograph, and the raw score. The raw score is a validated prognostic score for patients with smear-positive pulmonary TB [8].

We also collected data on the following “facility and physician-related factors”: year of hospital visit, time of arrival (day or night), weekday or weekend arrival, the attending ER physician, medical care provided by the resident on arrival, medical care provided by male physician on arrival, and whether transferred to the hospital by ambulance.

### Statistical analysis

We aimed to evaluate factors associated with delayed isolation of smear-positive pulmonary TB patients. The primary outcome measure was the duration day from hospital visit to the isolation of the patient with TB. Day-to-isolation analysis, based on the Cox proportional hazard model, was used to analyze factors associated with delay to the isolation of pulmonary TB patients. In a univariate model, the “patient-related factors” and “medical facility and physician-related factors” were evaluated as possible reasons for the delayed isolation of active pulmonary TB, based on log-rank test or univariate Cox hazard test, as appropriate. Significant factors in the univariate model were analyzed using the multivariate Cox proportional hazard model. A two-tailed *p*-value < 0.05 was considered statistically significant. All data analyses were undertaken using the SPSS Statistics version 21 J (IBM, Tokyo, Japan). This study was approved by the Institutional Review Board of the hospital (No. TGE00667-024).

## Results

Sixty-nine patients with smear-positive pulmonary TB were enrolled. Base-line characteristics and univariate analysis were included in Table 1. The mean age of patients was 81 years, with 18 (26.1%) female patients. All patients were hospitalized and isolated for infection control. Two patients were discharged home, 55 were transferred to other hospitals for the treatment of TB, and 12 died in our hospital. The median length of hospital stay was 7 days with interquartile range (IQR), 5–18 days.

**Table 1:** Clinical characteristics and day-to-isolation univariate analysis (*N* = 69)

Characteristic	No.(%) or mean ± SD	Day-to-isolation (95% CI)	*p*-value
Patient factor			
Age (years)	81.1 ± 10.0		0.827
Male gender-no.(%)	51 (73.9)	8.2 (3.90–12.41)	0.009*
Female gender-no.(%)	18 (26.1)	1.8 (0.70–2.86)	
Smoking-no.(%)	40 (58.0)	6.6 (3.16–10.09)	0.585
Non-smoking-no.(%)	29 (42.0)	6.3 (0.24–12.38)	
Alcoholism-no.(%)	4 (5.8)	1.5 (0.52–2.48)	0.217
Non-alcoholism-no.(%)	65 (94.2)	6.8 (3.40–10.21)	
Welfare recipient-no.(%)	4 (5.8)	1.3 (0.76–1.74)	0.172
Non-welfare recipient-no.(%)	65 (94.2)	6.8 (3.41–10.22)	
Dependent ADL-no.(%)	12 (17.4)	11.5 (2.67–20.33)	0.129
Partially dependent ADL-no.(%)	20 (29.0)	8.7 (0.08–17.22)	
Independent ADL-no.(%)	37 (53.6)	3.7 (1.28–6.13)	
Past history of tuberculosis-no.(%)	28 (40.6)	3.0 (1.44–4.49)	0.073
Non-past history of tuberculosis-no.(%)	41 (59.4)	8.9 (3.69–14.12)	
Contact with tuberculosis patients-no.(%)	7 (10.1)	3.3 (0.01–6.56)	0.519
Non-contact with tuberculosis patients-no.(%)	62 (89.9)	6.9 (3.30–10.41)	
Diabetes mellitus-no.(%)	25 (37.3)	3.8 (1.19–6.49)	0.248
Non-diabetes mellitus-no.(%)	42 (60.9)	8.3 (3.31–13.31)	
Other lung disease-no.(%)	18 (26.1)	5.3 (0.73–9.83)	0.864
Non-other lung disease-no.(%)	51 (73.9)	6.9 (2.86–10.99)	
Gastric resection-no.(%)	8 (11.6)	4.8 (0.00–11.02)	0.593
Non-gastric resection-no.(%)	61 (88.4)	6.7 (3.16–10.28)	
Human immunodificiency virus infection -no.(%)	1 (1.4)	1.0 (1.00–1.00)	0.366
Non-human immunodificiency virus infection-no.(%)	68 (98.6)	6.6 (3.31–9.84)	
Preceding newer quinolone antibiotics-no.(%)	6 (8.7)	23.0 (0.00–51.14)	0.027*
Non-preceding newer quinolone antibiotics-no.(%)	63 (91.3)	4.9 (2.77–7.07)	
Steroid use-no.(%)	15 (21.7)	6.5 (0.38–12.55)	0.923
Non-steriod use-no.(%)	54 (78.3)	6.5 (2.72–10.28)	
Chronic cough-no.(%)	19 (27.5)	2.3 (0.81–3.82)	0.023*
Absence of chronic cough-no.(%)	50 (72.5)	8.1 (3.74–12.42)	
Appetite loss-no.(%)	48 (70.6)	8.5 (3.96–13.00)	0.037*
Absence of appetite loss-no.(%)	20 (29.0)	2.0 (1.36–2.64)	
BMI (kg/m^2^)	19.0 ± 3.3		0.213
BT on arrival (°C)	37.2 ± 1.8		0.335
sBP on arrical (mmHg)	123.5 ± 30.3		0.768
Oxygen requirement-no.(%)	25 (36.2)	8.2 (0.89–15.51)	0.555
Non-oxygen requirement-no.(%)	44 (63.8)	5.5 (2.60–8.44)	
Haemoglobin level (g/dL)	11.9 ± 1.9		0.783
Serum albumin level (g/dL)	2.9 ± 0.6		0.348
CRP level (mg/dL)	9.3 ± 5.6		0.748
Cavitary lesion on the chest radiograph-no.(%)	15 (21.7)	1.3 (1.04–1.50)	0.005*
Non-cavitary lesion on the chest radiograph-no.(%)	54 (78.3)	7.9 (3.91–11.98)	
Upper zone lesion on the chest radiograph-no.(%)	36 (52.2)	3.3 (1.33–5.17)	0.015*
Lesion located other than in the upper zone on the chest radiograph-no.(%)	33 (47.8)	10.0 (3.80–16.26)	
Disseminated lesion on the chest radiograph-no.(%)	32 (46.4)	3.3 (0.72–5.91)	0.028*
Non-disseminated lesion on the chest radiograph-no.(%)	37 (53.6)	9.2 (3.79–14.70)	
Raw score (from −30 points to 60 points)	29.2 ± 18.0		0.973
Medical facility and doctor factor			
Year of hospital visit (from 2008 to 2016)	2012.4 ± 2.6		0.151
Night-time arrival-no.(%)	27 (39.1)	8.7 (1.87–15.47)	0.248
Day-time arrival-no.(%)	42 (60.9)	5.1 (2.11–8.08)	
Weekend arrival-no.(%)	13 (18.8)	3.8 (1.06–6.48)	0.517
Weekday arrival-no.(%)	56 (81.2)	7.1 (3.22–11.03)	
Medical care provided by resident-no.(%)	27 (39.1)	7.1 (0.72–13.58)	0.844
Medical care provided by non-resident-no.(%)	42 (60.9)	6.1 (2.71–9.44)	
Medical care provided by male doctor-no.(%)	60 (87.0)	5.9 (2.64–9.20)	0.407
Medical care provided by female doctor-no.(%)	9 (13.0)	10.3 (0.00–22.07)	
Transferred to the hospital by ambulance-no.(%)	39 (56.5)	7.9 (2.74–13.16)	0.523
Transferred to the hospital by non-ambulance-no.(%)	30 (43.5)	4.6 (1.63–7.57)	
Attending ER physician-no.(%)	55 (79.7)	6.3 (2.53–10.05)	0.580
Attending non-ER physician-no.(%)	14 (20.3)	7.3 (1.27–13.30)	

Raw score is tuberculosis prognostic score, which is calculated as follows:

age(years) + (oxygen requirement, 10 points) − 20 × albumin (g/dl) + (ADL: independent, 0 point; semi-dependent, 5 points; totally dependent, 10 points)

Resident indicate physician who was graduated within two years

Based on logrank test or cox hazard crude model, where appropriate, *p < 0.05

*CI* confidence interval, *SD* = standard deviation, *ADL* activities of daily living, *BMI* body mass index, *BT* body temperature, *sBP* systolic blood pressure, *CRP* C-reactive protein, *ER* emergency room

**Fig. 1:**
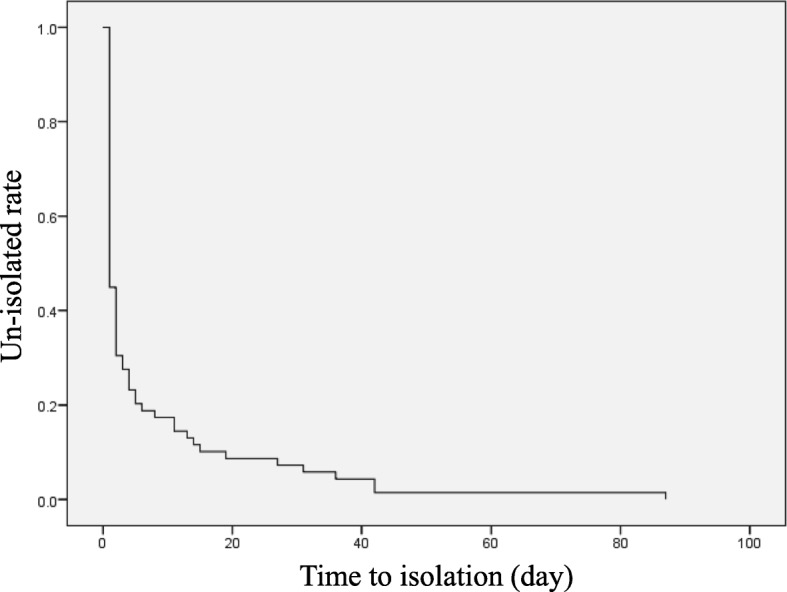
Kaplan-Meier curve of the time from admission to isolation of pulmonary TB patients. The median time to the isolation of pulmonary TB patients was 1 day with interquartile range, 1–4 days

The median day to the isolation of pulmonary TB patients was 1 day with IQR, 1–4 days. Figure 1 showed that over 60% patients isolated within 1 day. However, approximately 10% patients isolated over 10 days (Fig. 1). On univariate analysis, male gender (*p* = 0.009), prior treatment with newer quinolone antibiotics (*p* = 0.027), absence of chronic cough (*p* = 0.023), appetite loss (*p* = 0.037), non-cavitary lesion on the chest radiograph (*p* = 0.005), lesion located other than in the upper zone on the chest radiograph (*p* = 0.015), and non-disseminated lesion on the chest radiograph (*p* = 0.028) were significant factors associated with a delayed isolation of pulmonary TB patients (Table 1).

On multivariate analysis using Cox hazard model, male gender (hazard ratio [HR], 0.47; 95% confidence interval [CI], 0.25 to 0.89; *P* = 0.02), absence of chronic cough (HR, 0.52; 95% CI, 0.28 to 0.95; *P* = 0.033), and non-cavitary lesion on the chest radiograph (HR, 0.46; 95% CI, 0.23 to 0.92; *P* = 0.028) were significantly associated with delayed isolation of TB patients (Table 2).

**Table 2:** Associated factors with delayed isolation among patients with smear positive tuberculosis

Variable	Adjusted HR	95% CI for HR	*P* value
Male gender	0.47	(0.249 to 0.888)	0.020*
Appetite loss	0.63	(0.351 to 1.133)	0.123
Absence of chronic cough	0.52	(0.279 to 0.948)	0.033*
Preceding newer quinolone antibiotics	0.50	(0.190 to 1.290)	0.150
Non-cavitary lesion on the chest radiograph	0.46	(0.233 to 0.922)	0.028*
Non-upper zone lesion on the chest radiograph	0.64	(0.367 to 1.124)	0.120
Non-disseminated lesion on the chest radiograph	0.82	(0.479 to 1.404)	0.470

Multivariable Cox proportional hazards model including all factors *p* < 0.05 in univariate analysis

*HR* hazard ratio, *CI* confidence interval, *CRP* C-reactive protein

*p < 0.05

**Fig. 2:**
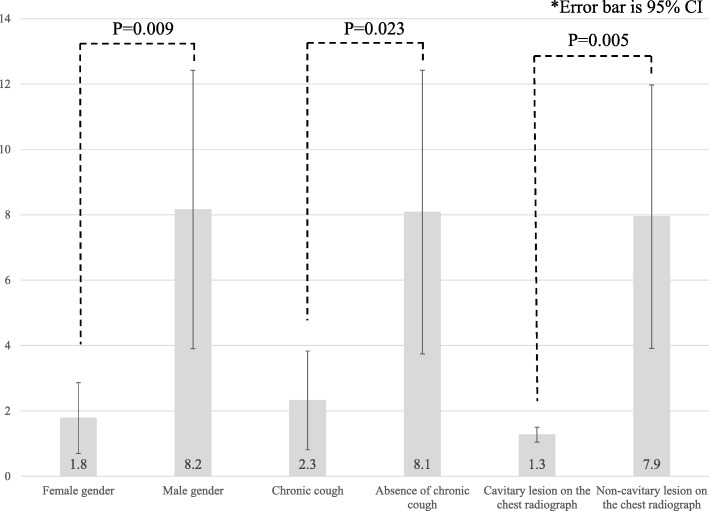
Grouped bar graphs indicating day-to-isolation of significant factors associated with delayed isolation of pulmonary TB patients

Figure 2 showed intergroup comparisons of day-to-isolation among significant factors associated with delayed isolation of pulmonary TB patients in the multivariate analysis.

## Discussion

Our study revealed that male gender, absence of chronic cough, and non-cavitary lesion on the chest radiograph were associated with delayed isolation of active pulmonary TB patients.

A non-cavitary lesion on the chest radiograph and absence of chronic cough are factors consistent with previous reports of diagnostic delay in cases of pulmonary TB; our findings of association of male gender with diagnostic delay is contrary to the findings of previous studies. Chronic cough is an important symptom for the clinician to consider the diagnosis of pulmonary TB from the clinical history. Moreover, a previous report of in-hospital diagnostic delay from Southern Taiwan revealed non-cavitary lesion on the chest radiograph as an independent risk factor [14], similar to our finding. A chest radiograph is commonly employed and easy to perform; presence of a cavitary lesion is critical in the early detection of pulmonary TB. In previous systematic reviews and meta-analyses, female gender has been shown to be a risk factor for delayed diagnosis of pulmonary TB [11, 12]; there are no previous reports of male gender being associated with delay in isolation of pulmonary TB patients.

We speculate that the delay in isolation associated with male gender, absence of chronic cough and non-cavitary lesion on the chest radiograph, may be related to the advanced age of our patients (mean age: 81.1 years). Younger patients with smear-positive TB were referred to other hospitals with special wards, resulting in an increase in elderly patients with TB in our hospital. Elderly patients with TB can manifest with unusual features that may be confused with aspiration pneumonia [17], commonly observed in this population [18]. The typical symptoms of aspiration pneumonia are fever, cough with sputum, and dyspnea; however, non-specific symptoms including loss of consciousness and appetite loss are also important. Thus, the symptoms of pulmonary TB and aspiration pneumonia are similar. Moreover, aspiration pneumonia, a common disease in an aging society, can co-exist in patients with active pulmonary TB and has drawn increasing attention recently [19]. Although the incidence of active pulmonary TB has decreased, the number of elderly patients has increased in Japan [20]. There may be a lack of widespread awareness among healthcare workers, including physicians, of this development. This may have led to failure to consider pulmonary TB in elderly patients. Besides, the symptoms of pulmonary TB can mimic aspiration pneumonia in the elderly.

Although previous studies have described factors associated with delayed diagnosis of pulmonary TB [11–13, 15, 21–27], we focused on factors that delayed isolation, especially among elderly patients admitted to hospital. To the best of our knowledge, ours is the first study that deals with delayed isolation of pulmonary TB patients in an aging society [28, 29]. According to nation-wide Japanese data, the number of patients over 70 years with smear-positive pulmonary TB has increased by 2.5 times from 1980 to 2000 [20, 30]. In an aging society, the number of older patients with smear-positive pulmonary TB may have increased in community-based acute care hospitals. However, there are few reports of elderly patients with pulmonary TB. Thus, our results may be of relevance in an aging society.

There are several limitations to our study. First, ours was based on a single center cohort study with a small sample size. The clinical ability of our residents was variable, depending on their level of training and experience, which may have led to bias. In our study model, a *p* < 0.05 was used as the cutoff level. This strict criteria though can prematurely exclude relevant variables, which may lead to selection bias. Second, we are unable to establish a causal relationship in this retrospective observational study. Further prospective studies are required to confirm such an association. Third, as it was a hospital-based study, we could not analyze factors involving delay from symptoms onset to hospital visit, reported as “patient delay” in a previous study [16]. Fourth, the present study may not include all in-hospital patients with smear-positive pulmonary TB, because there can be presence of the patients who discharged or died before the investigation of sputum or gastric smear.

Future research needs to be directed to understanding factors associated with a delayed diagnosis of active pulmonary TB; this information may help with earlier isolation of TB patients, improve infection control, and reduce adverse outcomes. Therefore, multi-center, prospective studies are required, across different population groups, in countries with aging populations, to corroborate our results.

## Conclusion

Isolation of active pulmonary TB patients was delayed in male patients, patients without chronic cough, and patients with non-cavitary lesions on the chest radiograph. Elderly TB patients can present with atypical manifestations that mimic other diseases. Thus, physicians need to have a heightened awareness of active pulmonary TB, especially among elderly patients; this may prevent spread of infection within the hospital.

## Abbreviations

CI: Confidence interval
ER: Emergency room
HR: Hazard ratio
IQR: Interquartile range
TB: Tuberculosis
